# Vietnamellidae (Insecta, Ephemeroptera) of Thailand

**DOI:** 10.3897/zookeys.902.46844

**Published:** 2020-01-13

**Authors:** Chonlakran Auychinda, Michel Sartori, Boonsatien Boonsoong

**Affiliations:** 1 Animal Systematics and Ecology Speciality Research Unit (ASESRU), Department of Zoology, Faculty of Science, Kasetsart University, Bangkok 10900, Thailand Kasetsart University Bangkok Thailand; 2 Museum of Zoology, Palais de Rumine, Place Riponne 6, CH-1005 Lausanne, Switzerland Museum of Zoology Lausanne Switzerland; 3 Department of Ecology and Evolution, Lausanne University, CH-1015 Lausanne, Switzerland Lausanne University Lausanne Switzerland

**Keywords:** COI, Ephemerelloidea, mayfly, phylogeny, *
Vietnamella
*

## Abstract

The genus *Vietnamella* Tshernova, 1972 is investigated in detail for the first time in Thailand. As a consequence, four species are recognized, namely *Vietnamella maculosa***sp. nov.**, *Vietnamella thani* Tshernova, 1972, *Vietnamella* sp. B and *Vietnamella* sp. C. Herein, larvae and eggs of *V. maculosa***sp. nov.** are described and reported from Chiang Rai Province. The larva of *Vietnamella* sp. B from Tak Province is also described, but not named due to insufficient material, and the imaginal stages and eggs of *V. thani* Tshernova, 1972 are described and presented for the first time. Our morphological evidence is supported with COI data. The phylogeny showed that four different lineages of the genus *Vietnamella* occur in Thailand, one of them, viz., *Vietnamella* sp. C, only known from a couple of COI sequences retrieved from the Barcode of Life Data System (BOLD). Diagnoses for all known Oriental species are also presented.

## Introduction

The monogeneric family Vietnamellidae was originally established by Tshernova (1972) [type species: *Vietnamella thani* Tshernova, 1972] based on larval specimens. The status of the family and the taxonomic history of the genus *Vietnamella* Tshernova, 1972 are reviewed by Jacobus et al. (2005) and Hu et al. (2017) respectively. Nowadays, three species have been described from the Oriental region. They are *V. ornata* (Tshernova, 1972), *V. sinensis* (Hsu, 1936) [=*V. dabieshanensis* You & Su, 1987, *V. qingyuanensis* Zhou & Su, 1995, *V. guadunensis* Zhou & Su, 1995], and *V. thani* (Hu et al. 2017). The larval and imaginal stages of *V. sinensis* have been described, whereas *V. thani* is only known from the larval stage and *V. ornata* (Tshernova, 1972) only from the sub-imaginal stage. Recently, *Vietnamella* sp. A was described from India, based on its larvae which present the unique character of outer projections on the head with serrations (Selvakumar et al. 2018). However, the authors were unable to confirm species identification using current morphological and molecular data because the larval morphology of *V. ornata* has never been described, and the distribution of *Vietnamella* sp. A is close to the type locality of *V. ornata* (Selvakumar et al. 2018).

The genus *Vietnamella* is endemic in the Oriental region and is distributed in China, Thailand, India and Vietnam (Tshernova 1972; Jacobus et al. 2005; Hu et al. 2017; Selvakumar et al. 2018). In Thailand, *Vietnamella* has never been reported at the species level. Here, we review the species of *Vietnamella* in Thailand and describe a new species in the genus and the imaginal stages of *V. thani* based on reared specimens. A distribution map and mitochondrial COI sequence data are also provided.

## Materials and methods

### Ethics statement

The present study was approved by the ethics committee of Kasetsart University (approval no. ACKU61-SCI-029) for rearing and collecting the mayfly specimens.

### Morphological observations

The vietnamellid larvae were collected from fast-flowing areas of streams in northern and western Thailand. The imagoes were reared from mature larvae in the laboratory. Measurements (mm) and photographs were taken using a Nikon SMZ800 stereoscopic microscope and a Canon EOS 6D camera with MP-E 65 mm macro lens. For scanning electron microscopy (SEM), eggs were dried, coated with gold and observed with a FEI Quanta 450 SEM instrument. Final plates were prepared with Adobe Photoshop CC 2017. The specimens are deposited in the collection of the Zoological Museum at Kasetsart University in Bangkok, Thailand (**ZMKU**) and at the Museum of Zoology in Lausanne, Switzerland (**MZL**). The distribution map was generated via the Simple Mapper website using GPS coordinates (http://www.simplemappr.net).

### Molecular analysis

The collected specimens were fixed in absolute ethanol and preserved under refrigeration for description and DNA extraction. Collection details of the specimens of the three species used for the DNA experiment are shown in Table 1. Part of the specimens was extracted by using non-destructive methods. Total DNA was extracted using a genomic DNA purification kit (NucleoSpin, Macherey-Nagel, Germany) following the manufacturer’s protocol. A fragment of the mitochondrial cytochrome oxidase I (COI) was amplified (658 bp) using the primers LCO1490 (5'-GGT CAA ATC ATA AAG ATA TTG G-3') and HCO2198 (5'-TAA ACT TCA GGG TGA CCA AAA AAT CA-3'), designed by Folmer et al. (1994). Polymerase chain reaction (PCR) conditions were as follows: a 25 μl final total volume containing 12 μl of PCR Master Mix solution, 1.5 μl (10 μM) of each primer, 5 μl of DNA and 5 μl of sterile water. PCR was performed as follows: 5 minutes at 94 °C, then 30 seconds at 94 °C, 30 seconds at 48 °C and 60 seconds at 72 °C (40 cycles), and a final elongation step at 72 °C for 10 minutes (Gattolliat et al. 2015). Purification and sequencing were conducted by Macrogen, Inc. (South Korea). Sequence alignment and editing were performed using ClustalW. The phylogenetic tree was analysed by Bayesian inference using MrBayes. The evolution model obtained was General Time Reversible Model and Gamma distributed with invariant sites (GTR+G+I). Nucleotide sequences obtained in this study have been deposited in GenBank database. Other analysed mayfly sequences were obtained from the Barcode of Life Data System (BOLD): *Vietnamella* sp. C (THMAY031-09.COI-5P, THMAY148-12.COI-5P and THMAY149-12.COI-5P); and GenBank: *Vietnamella* sp. 1 (KM207084.1; KM244655.1) and *V. dabieshanensis* (HM067837.1). Other Ephemerelloidea COI sequences from GenBank including *Dudgeodes palnius* (LC057264.1), *Teloganella indica* (LC057266.1), *Teloganopsis deficiens* (HQ958649.1) were added to the analysis. *Potamanthellus edmundsi* (MN186576) was used as an outgroup.

**Table 1.** Collection details of the sequenced specimens.

Species	Code	Collection locality	Collector	Date	GenBank Accession Number
*V. maculosa* sp. nov.	VmCR01	Chiang Rai	D. Chainthong	6-5-2019	MN510862
	VmCR02				MN510863
*Vietnamella* sp. B	VbTK01	Tak	A. Watcharangkool	12-1-2016	MN204621
*V. thani*	VtKN01	Kanchanaburi	B. Boonsoong	20-2-2016	MN204618
*V. thani*	VtKN02	Kanchanaburi	B. Boonsoong	20-2-2016	MN204619
*V. thani*	VtKN03	Kanchanaburi	B. Boonsoong	21-2-2016	MN204620
*V. thani*	VtPK01	Prachuap Khiri Khan	D. Chainthong	19-4-2019	MN318306

## Taxonomy

### Family Vietnamellidae Allen, 1984

#### Genus *Vietnamella* Tshernova, 1972

##### Vietnamella maculosa sp. nov.

Taxon classificationAnimaliaEphemeropteraVietnamellidae

85D69006-1E6F-57A2-A1B8-6602C0042EE5

http://zoobank.org/E75B83EC-A077-4533-83EC-224D5A26E1DA

**Figure 1.**
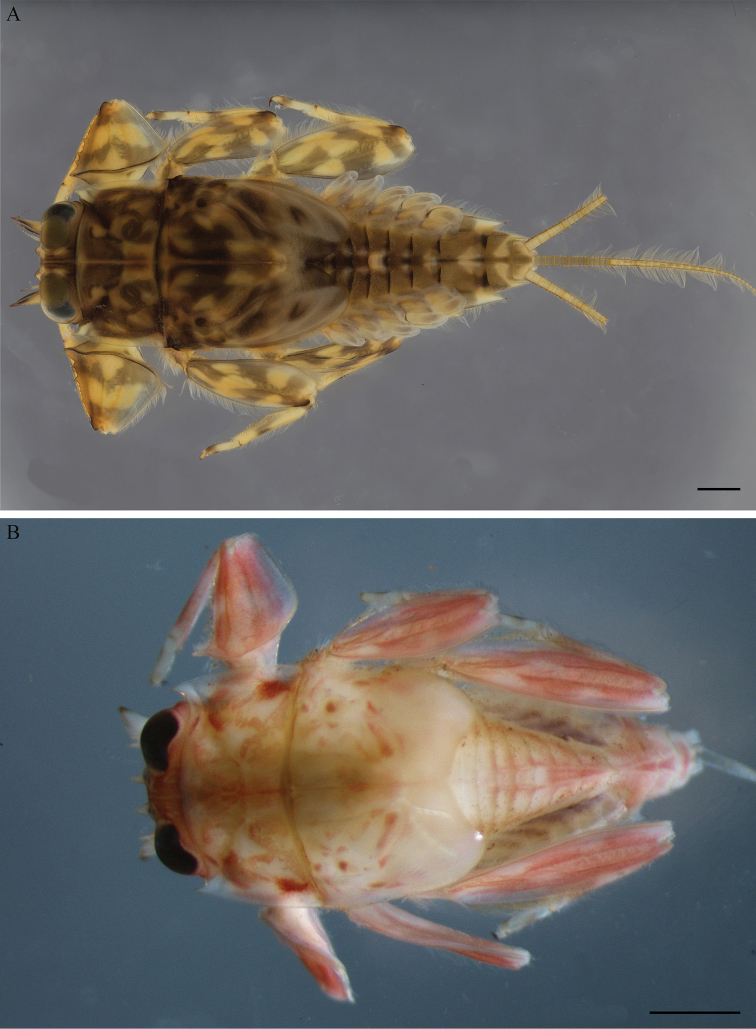
*Vietnamella* spp. **A***Vietnamella maculosa* sp. nov., habitus in dorsal view **B***Vietnamella* sp. B, habitus in dorsal view. Scale bars: 1 mm.

**Figure 2.**
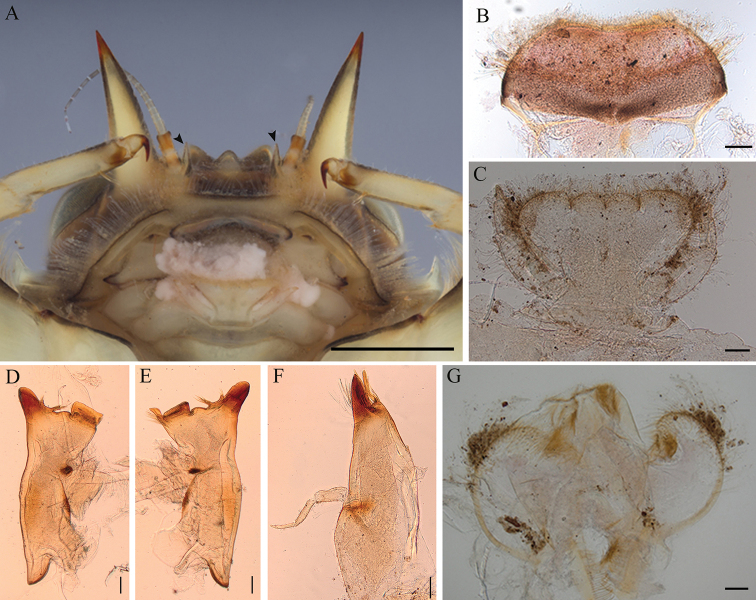
*Vietnamella maculosa* sp. nov. **A** ventral view of head with outer projection and small inner projection (arrow) **B** labrum **C** labium **D** left mandible **E** right mandible **F** maxilla **G** hypopharynx. Scale bars: 1 mm (**A**); 0.1 mm (**B–G**).

**Figure 3.**
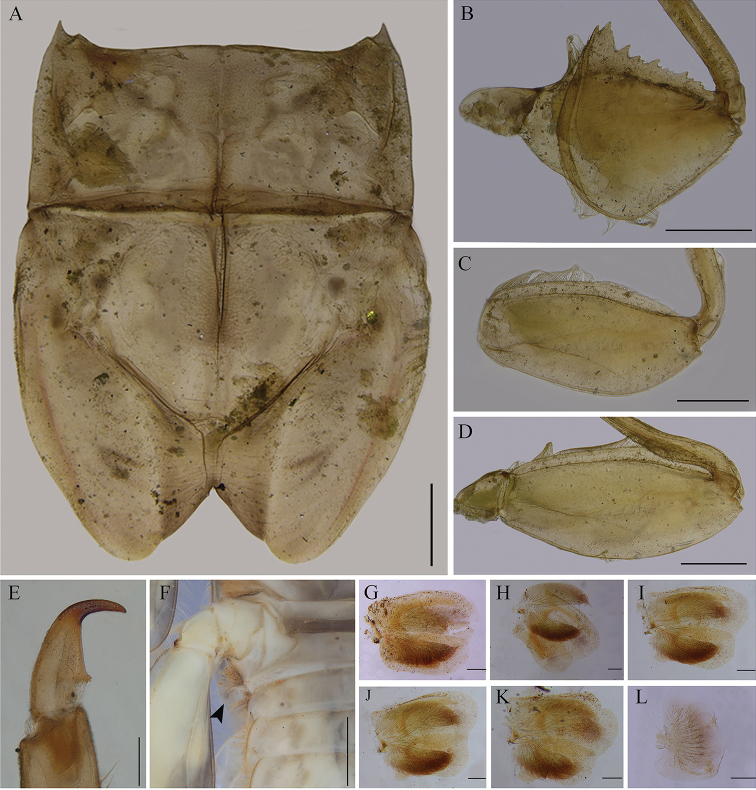
*Vietnamella maculosa* sp. nov. **A** thorax **B** forefemur **C** middle femur **D** hind femur **E** foreleg claw **F** first gills on segment I (arrow) **G** gill II **H** gill III **I** gill IV **J** gill V **K** gill V **L** gill VII. Scale bars: 1 mm (**A–D, F**); 0.2 mm (**G–L**); 0.1 mm (**E**).

**Figure 4.**
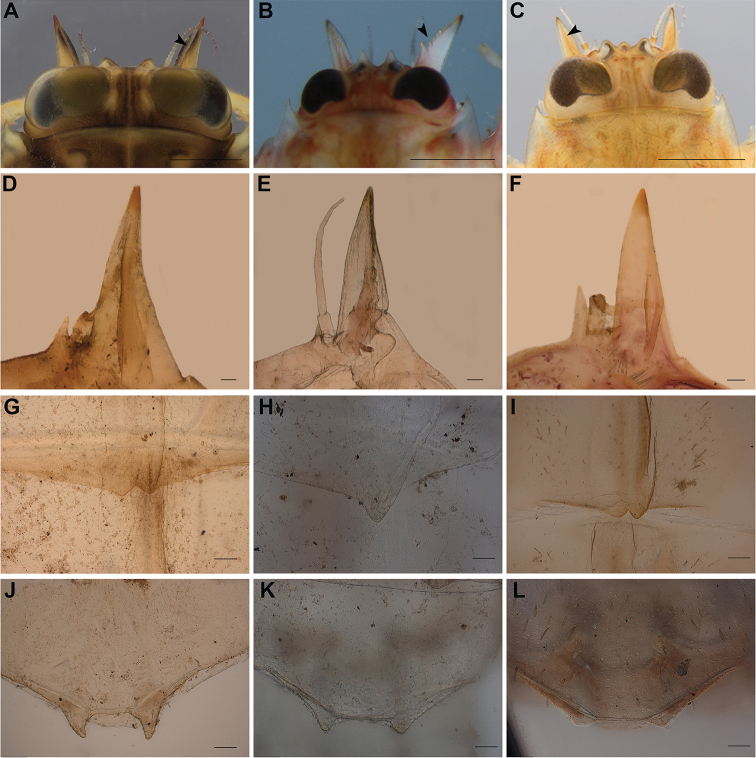
*Vietnamella* spp. *Vietnamella maculosa* sp. nov. (**A, D, G, J**): **A** outer projection on head (arrow) **D** inner and outer projections on head **G, J** tubercle on tergite VII and X; *Vietnamella* sp. B (**B, E, H, K**): **B** outer projection on head (arrow) **E** inner and outer projections with serration on head **H, K** tubercles on tergite VII and X; *Vietnamella thani* (**C, F, I, L**): **C** outer projection on head (arrow) **F** inner and outer projections on head **I, L** tubercles on tergite VII and X. Scale bars: 1 mm (**A–C**); 0.5 mm (**G**); 0.1 mm (**D–F, H, I**); 0.05 mm (**J–L**).

**Figure 5.**
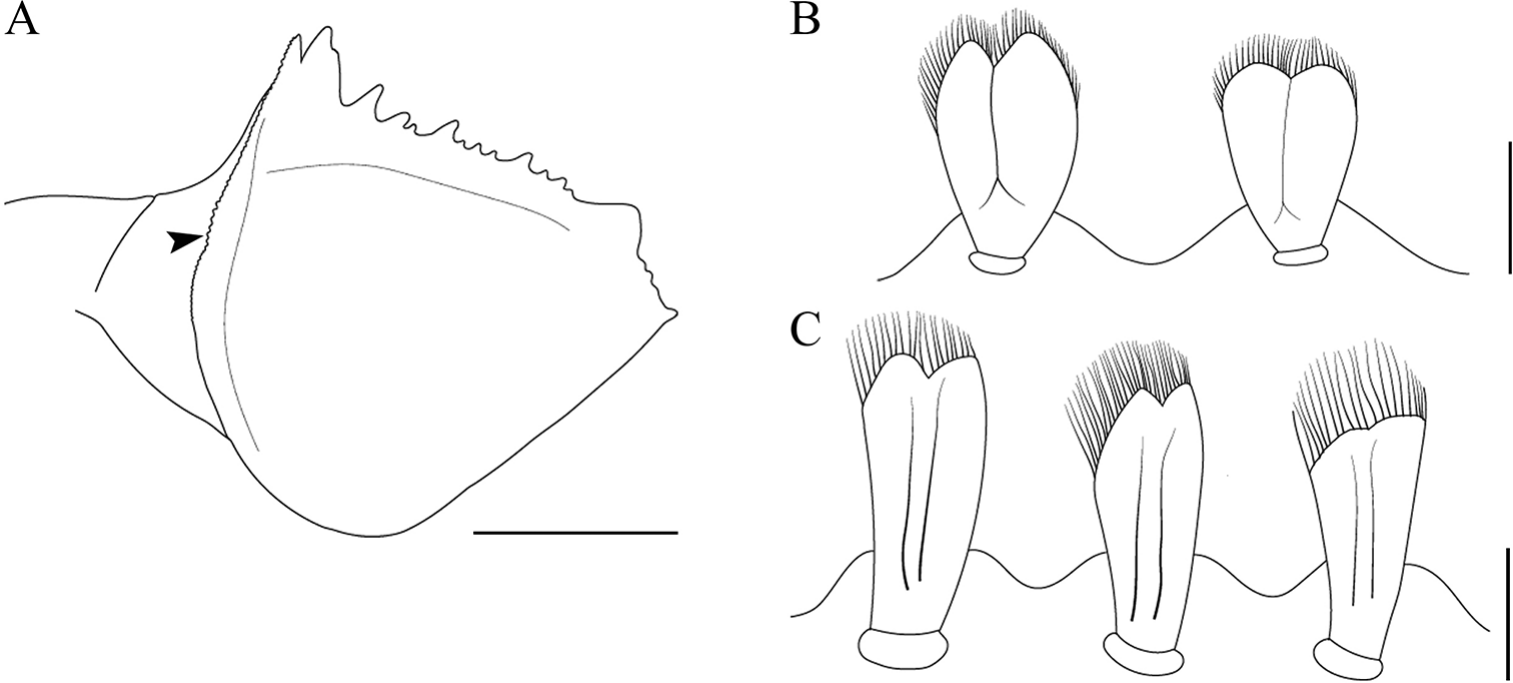
Forefemur and setae **A** forefemur of *Vietnamella maculosa* sp. nov. with serration of transverse ridge (arrow) **B** setae on transverse ridge of *Vietnamella maculosa* sp. nov. **C** setae on transverse ridge of *V. thani*. Scale bars: 1 mm (**A**); 0.01 mm (**B, C**).

**Figure 6.**
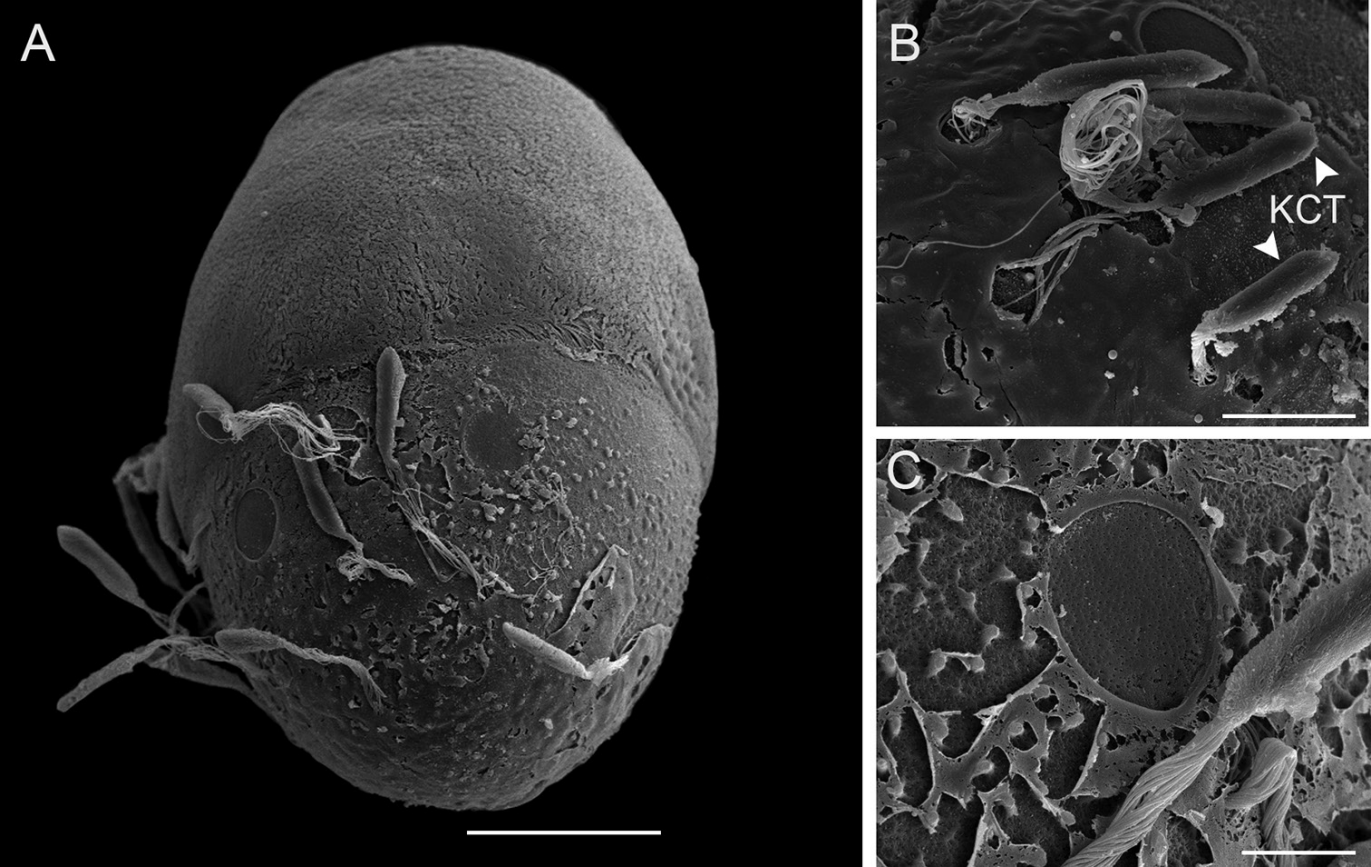
SEM of egg structure of *Vietnamella maculosa* sp. nov. **A** overview **B** detail of Knob Terminated Coiled Thread (KCT) **C** micropyle and chorionic surface. Scale bars: 50 µm (**A**); 20 µm (**B**); 10 µm (**C**).

Figs 1A , 2A–G , 3A–L , 4A, D, G, J , 5A, B , 6A–C

###### Material examined.

***Holotype***: 1 male larva Thailand, Chiang Rai Province, Mueang Chiang Rai, Pong Phra Bat waterfall, 20°00'41.0"N, 99°48'15.0"E, 470 m, 6.V.2019, D. Chainthong leg. [ZMKU]. ***Paratypes***: 2 larvae same data as holotype; 1 larva on slide [ZMKU] and another in ethanol [MZL GBIFCH00673059]. Both paratypes were used for DNA extraction.

###### Description.

Mature larva (in alcohol, Fig. 1A). Body length 11.25 mm without cerci; cerci 4.8 mm; body brown with dark brown markings on thorax and femora.

***Head*.** Brown with a pair of occipital tubercles, a single sub-occipital tubercle medially; two projections below eyes; inner pairs of projections small, spine-like and sharp (Fig. 2A); outer pair large, triangular, cone shaped without any serrated spines (Fig. 4A, D). Labrum, similar to other vietnamellid mayflies, anterior half of dorsal surface and margins with relatively long setae, ventral surface with short setae (Fig. 2B). Labium, glossae width greater than length, glossae and paraglossae with dense setae on surface, setae on dorsal surface and margins longer; labial palpi three segmented, basal segment broader and longer than the second, apical segment very small; palpi with tiny setae (Fig. 2C). Left mandible, slender, a little concave at sub-median area; molar block-like shape with a tuff of short setae below inner molar margin (Fig. 2D). Right mandible, slender, slightly concave at sub-median area; molar block-like shape with a row of setae below inner molar margin (Fig. 2E). Maxillae slender; maxillary palpi three segmented, with tiny setae; length ratio from basal to apical = 1.3:1.2:1 (Fig. 2F). Hypopharynx, lingua square and superlinguae nearly round, with setae on surface (Fig. 2G)

***Thorax*.** Pronotum with small moderately sharp anterolateral projections, and slightly pointed protuberances below the anterolateral projection (Fig. 3A). Forefemur, strongly expanded with serrations or teeth projections on ventral margin (Fig. 3B); transverse ridge serrated with small rounded setae (Fig. 5A, B) and long thin setae near inner dorsal margin. Midfemur without any projection, expanded, dorsal margin convex apically and with a row of hair-like setae (Fig. 3C). Hindfemur without any projection, expanded, longer than midfemur, dorsal margin with a row of hair-like setae (Fig. 3D). All claws similar, strongly hooked with a single small denticle basally (Fig. 3E).

***Abdomen*.** Tergites I–X with a pair of median ridges or tubercles; posterolateral angles of tergites II–X extended into sharp projection; tergite VII with a pair of tubercles (Fig. 4G) and tergite X with well-developed a pair of tubercles (Fig. 4J); lateral margins of tergite with dense setae. Gill on segments I-VII: gill I finger-like with setae (Fig. 3F); gills on segments II–VI similar in structure, with dorsal and ventral lamellae, the latter further divided into two clusters, each with several smaller lobes (Fig. 3G–K); gill on segment VII small, with two lamellae but ventral lamella divided into three lobes (Fig. 3L). Caudal filaments with dense lateral setae on inner and outer margins of middle part.

***Eggs.*** (dissected from mature larva). Length 200 µm, width 144 µm; oval shape, chorionic surface with small protuberances, half of egg covered with helmet-shaped polar cap (Fig. 6A); rod shaped KCT (Knob Terminated Coiled Thread) around egg body (Fig. 6B); 2 or 3 tagenoform-type micropyles at centre (Fig. 6C).

###### Diagnosis.

The larva of *Vietnamella maculosa* sp. nov. is most similar to that of *V. thani* and *V. sinensis* in the lack of serrations on the outer projection on the head, but it can be separated from *V. thani* based on the following characteristics: i) the pattern of serration on the ventral margin of the forefemur, ii) the setae on the transverse ridge of the forefemur, iii) a well-developed pair of median ridge projections of tergite X. It can be separated from *V. sinensis* by the proportion of the maxillary palp on the second segment, which is slightly longer than on the third segment (1.2:1), whereas in *V. sinensis* it is clearly longer (1.6:1).

###### Remarks.

The larvae of *Vietnamella maculosa* sp. nov. have a unique colour pattern, with brown and dark brown banding spread over the body and femurs. However, this colour can change in intensity depending on the life stage and environment. Other species, like *V. thani*, have variation in body colour but do not have this pattern.

###### Etymology.

The name *maculosa* (Latin for having spot or macula), refers to the brown and dark brown banding of the species.

###### Distribution.

Chiang Rai Province

###### Adult.

Unknown

##### Vietnamella

Taxon classificationAnimaliaEphemeropteraVietnamellidae

sp. B

5A1E1E11-19A1-5999-92F3-2D48105F9AEA

Figs 1B , 4B, E, H, K

###### Material examined.

Thailand; 1 larva (immature) on slide, Tak Province, Mae Ra-Merng, 17°31'18.7248"N, 98°3'36.8064"E, 26.X.2015, A. Watcharangkool leg. (ZMKU).

###### Description.

Larva (in alcohol, Fig. 1B). Body length 6 mm without cerci; body reddish with dense setae lateral and on margin of head.

***Head*.** Reddish with a sharp pair of occipital tubercles and a single sub-occipital tubercle medially; two projections below eyes: inner pairs of projections small, spine-like and sharp; outer pair large, triangular, cone shaped with five unequal serrated spines (Fig. 4B, E), basal spine the largest. Labrum, similar to *V. thani* and *V. maculosa* sp. nov. Labium, glossae width greater than length, glossae and paraglossae with dense setae on surface, labial palpi three segmented, basal segment broader and longer than the second, apical segment small; palpi with tiny setae. Left mandible and right mandible slender, mostly similar to the other *Vietnamella* species. Maxillae slender, maxillary palpi three segmented with tiny setae, length ratio from basal to apical = 1.3:1:1. Hypopharynx, lingua and superlinguae nearly round with setae on surface.

***Thorax*.** Pronotum with small sharp anterolateral projections, slight protuberances below the anterolateral projections. Forefemur strongly expanded with serrations or teeth projections on ventral margin; transverse ridge serrated with small setae. Midfemur without any projection. Hindfemur without any projection, expanded, longer than midfemur; dorsal margin with a row of hair-like setae. All claws similar, with one small denticle basally.

***Abdomen*.** Tergite I–VI and VIII–X with pair of median ridges or tubercles progressively; tergite VII with a single tubercle (Fig. 4H); posterolateral angles of terga II–IX extended into sharp projection; tergite X with a pair of moderately-developed tubercles (Fig. 4K); lateral margins of tergite with dense setae. Gills on segments III–VI similar in structure with dorsal and ventral lamella, the latter further divided into two clusters, each with several smaller lobes; gill VII small, with two lamellae but ventral lamella divided into three lobes.

###### Diagnosis.

The larva of *Vietnamella* sp. B can be separated from those of other species based on the following characteristics: i) outer pairs of projections on the head are large and stout, triangular, and cone shaped, with five unequally serrated spines (one large spine + four small spines), and ii) the abdominal tergites II–IX have a pair of projections or tubercles at the posterolateral margin except for tergite VII which has only a single projection (Fig. 4H).

###### Remarks.

The larval description given herein agrees with larvae of other species belonging to the genus *Vietnamella*, including the presence of a pair of projections on the head, the expanded femur and the forefemur with serrations on the outer dorsal margin. The larva described here has serrated spines on the outer projection of the head that differ from the other valid species of *Vietnamella*.The outer serrated projection is similar to that of *Vietnamella* sp. A from India (Selvakuma et al. 2018), but it differs in the number and character of the outer projection spines (four equal serrated spines in *Vietnamella* sp. A, five unequal serrated spines in *Vietnamella* sp. B). Although *Vietnamella* sp. B was not a mature larva, the phylogenetic analysis showed it belonged to a clearly different clade and had a high genetic distance compared to the other species (Fig. 12; Table 2). Formal description of this species is pending more material.

**Table 2.** Pairwise genetic distances (COI) between species of *Vietnamella* using the Kimura 2-parameter.

Taxa	K2P genetic distances					
	1	2	3	4	5	6
1. *Vietnamella maculosa* sp. nov.						
2. *Vietnamella thani*	0.253					
3. *Vietnamella* sp. B	0.286	0.267				
4. *Vietnamella* sp. C	0.276	0.160	0.286			
5. *Vietnamella* sp. 1	0.254	0.224	0.278	0.242		
6. *Vietnamella dabieshanensis*	0.258	0.184	0.309	0.185	0.217	
7. *Potamanthellus edmundsi*	0.267	0.206	0.229	0.248	0.289	0.242

###### Distribution.

Tak Province.

###### Adult and egg.

Unknown.

##### Vietnamella thani

Taxon classificationAnimaliaEphemeropteraVietnamellidae

Tshernova, 1972

0B070CDE-368F-5605-BB9B-A3894D30232E

**Figure 7.**
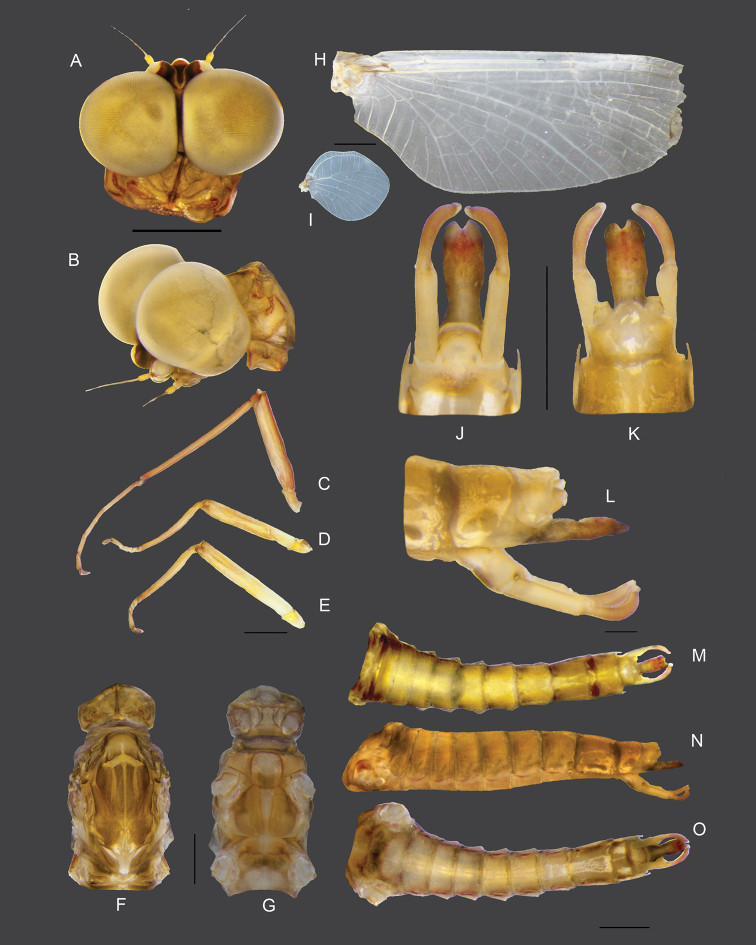
Male imago of *Vietnamella thani***A** dorsal view of head **B** lateral view of head **C** foreleg **D** middle leg **E** hindleg **F, G** dorsal and ventral view of thorax **H** forewing **I** hindwing **J** ventral view of genitalia **K** dorsal view of genitalia **L** lateral view of genitalia **M** dorsal view of abdomen **N** lateral view of abdomen **O** ventral view of abdomen. Scale bars: 1 mm.

**Figure 8.**
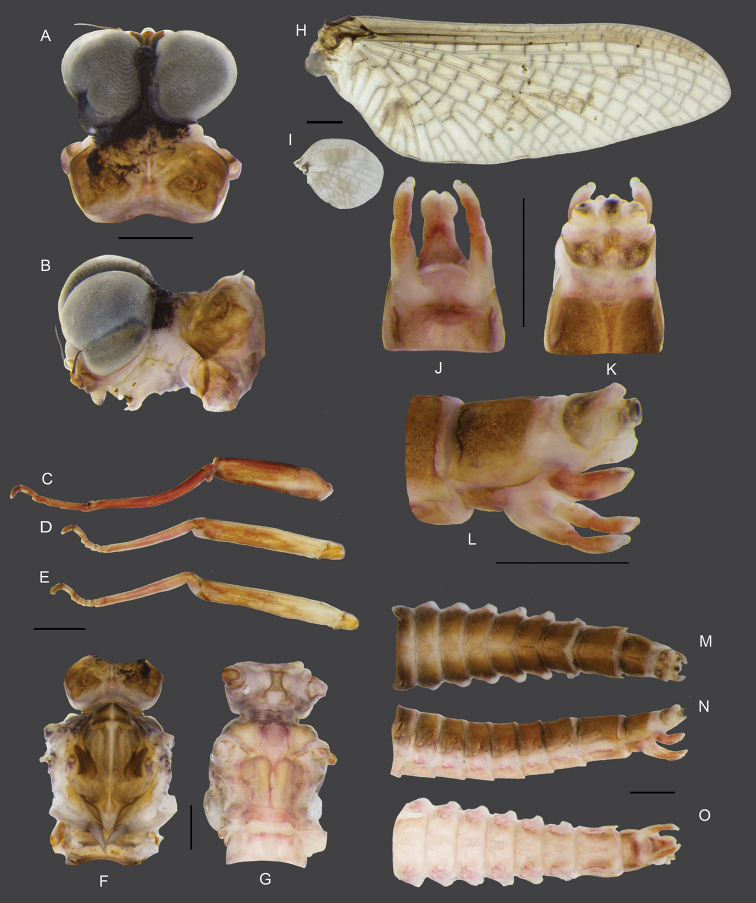
Male subimago of *Vietnamella thani***A** dorsal view of head **B** lateral view of head **C** foreleg **D** middle leg **E** hindleg **F, G** dorsal and ventral view of thorax **H** forewing **I** hindwing **J** ventral view of genitalia **K** dorsal view of genitalia **L** lateral view of genitalia **M** dorsal view of abdomen **N** lateral view of abdomen **O** ventral view of abdomen. Scale bars: 1 mm.

**Figure 9.**
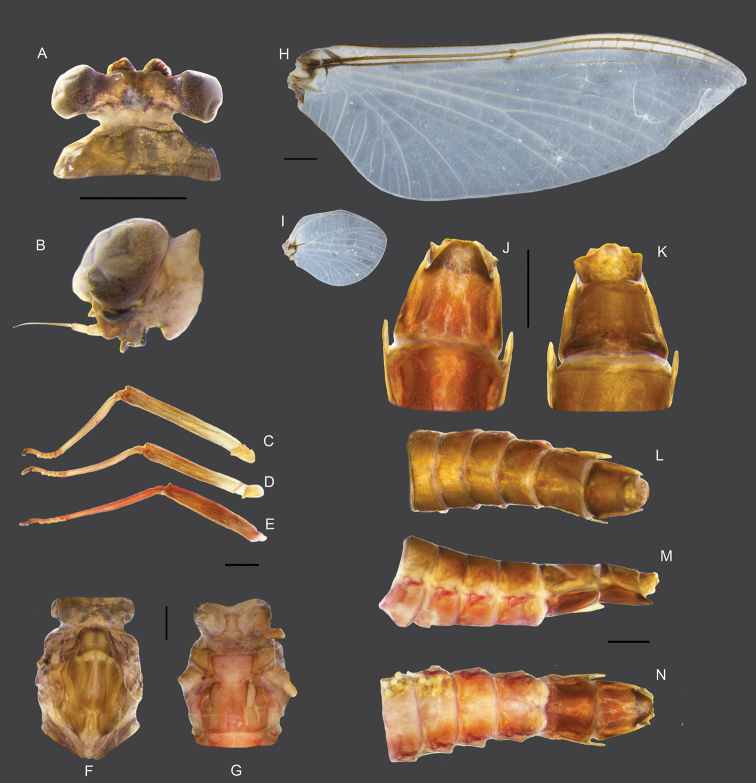
Female imago of *Vietnamella thani***A** dorsal view of head **B** lateral view of head **C** foreleg **D** middle leg **E** hindleg **F, G** dorsal and ventral view of thorax **H** forewing **I** hindwing **J, K** ventral and dorsal view of genitalia **L** dorsal view of abdomen **M** lateral view of abdomen **N** ventral view of abdomen. Scale bars: 1 mm.

**Figure 10.**
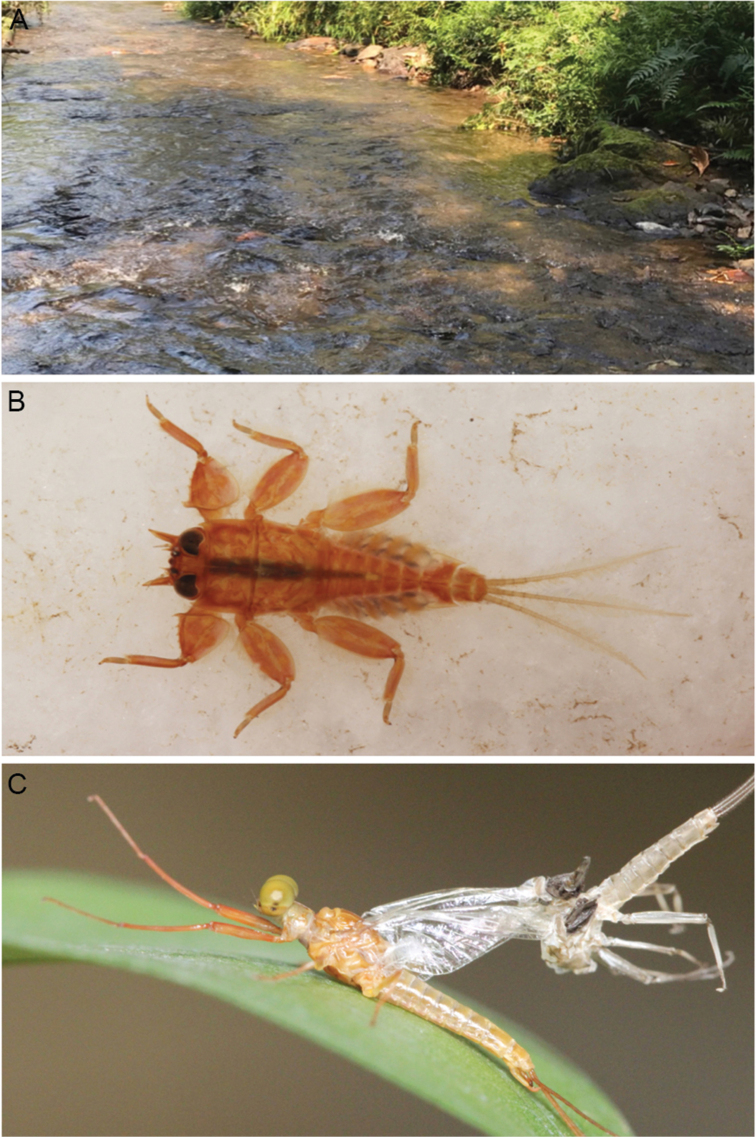
*Vietnamella thani***A** habitat **B** habitus of larva **C** habitus of male imago (incomplete molting).

**Figure 11.**
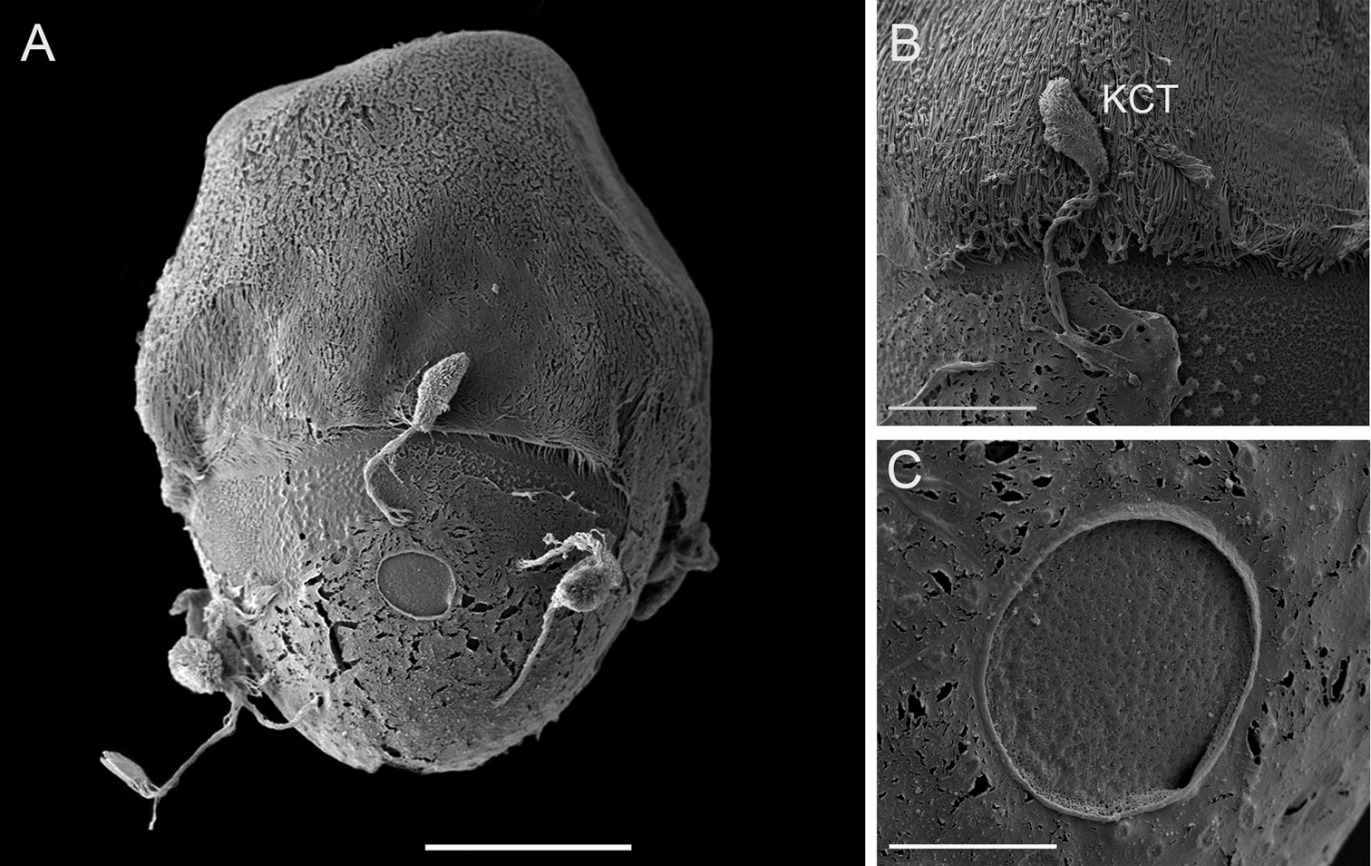
SEM of egg structure of *Vietnamella thani***A** overview **B** detail of Knob Terminated Coiled Thread (KCT) and chorionic surface **C** micropyle. Scale bars: 50 µm.

Figs 4C, F, I, L , 5C , 7A–O , 8A–O , 9A–N , 10B, C , 11A–C

Vietnamella thani Tshernova, 1972: 604–614, fig. 4 (orig.); Hu et al. 2017: 381–390, fig. 7 (distribution).

###### Material examined.

Thailand; Kanchanaburi Province, Thong Pha Phum, Huai Pak Kok, 14°35'01.4"N, 98°34'54.0"E, 161 m, 15.X.2015, 1 larva; 20.II.2016, 1 larva; 21.II.2016, 3 larvae, 1 female imago; 31.I.2019, 9 larvae, 1 male subimago (reared), 1 male imago (reared). Huai Khayeng, Ban Prachum Mai, 14°39'34.0"N, 98°32'02.0"E, 233 m, 20.II.2016, 4 larvae, [ZMKU]; 13.XII.2014, 1 larva, all B. Boonsoong leg; 15.X.2015, 3 larvae, B. Boonsoong & M. Sartori leg. [MZL]. Prachuap Khiri Khan Province, Kui Buri, Huai Samrong, 12°03'55.0"N, 99°37'38.0"E, 103 m, 11 larvae, D. Chainthong leg. [ZMKU].

###### Diagnosis.

The larva of *Vietnamella thani* can be distinguished from those of other *Vietnamella* based on the following characteristics: i) outer pairs of projections on the head are long, triangular, and cone shaped without serrated spines; ii) the first and second segments of the maxillary palpi have an equal length ratio; iii) the forefemur has a serrated transverse ridge with spatulate setae (Fig. 5C); and iv) the abdominal tergite X either lacks or has a poorly-developed pair of tubercles (Fig. 4L).

###### Description of imagoes.

**Male imago** (in alcohol, Figs 7, 10C). ***Head*.** Eyes rounded with ventral part brown-yellowish and dorsal part yellowish (Fig. 7A, B). ***Thorax*.** Forelegs (8.07 mm), length ratio of femur and tibia = 1:1.45; length ratio of four tarsal segments is 3:2:1.5:1 (Fig. 7C). Midlegs (4.76 mm), length ratio of femur and tibia = 1.2:1; length ratio of four tarsal segments is 1:1:1:3 (Fig. 7D). Hindlegs (4.97 mm), length ratio of femur and tibia = 1.4:1; length ratio of four tarsal segments is 1:1:1:4 (Fig. 7E). Mesonotum with a notable median longitudinal suture, two medioparapsidal sutures (Fig. 7F). Mesosternum with a square basisternum, broad furcasternum (Fig. 7G). Forewings, numerous crossviens. MA forked middle of wing, MP forked basally, three intercalaries between MP1 and MP2; CuA and CuP adjacent at base; cubital field with three bifurcate veins arising from CuA (Fig. 7H). Hind wings rounded, leading margin slightly concave, with clear crossveins; seven crossveins with one bifurcate between Sc and RA; three crossveins between MA and MP (Fig. 7I). ***Abdomen*.** Genitalia with three-segmented forceps (1.2 mm), first segment = 0.6 mm, second segment = 0.45 mm, apical segment = 0.15, small and nearly rounded; penes (0.76 mm) totally fused with a shallow median cleft; subgenital plate slightly convex (Fig. 7J–L). Abdominal segment IX with lateral projection, white stripe on sternites VII–IX (Fig. 7M–O).

**Male subimago.** (in alcohol, Fig. 8). ***Head*.** Eyes rounded; dorsal part dark grey; ventral part brown (Fig. 8A, B). ***Thorax*.** Forelegs (6.38 mm), length ratio of femur and tibia = 1:1.1; length ratio of four tarsal segments is 1.6:1:1:1.3 (Fig. 8C). Midlegs (5.41 mm), length ratio of femur and tibia = 1.4:1; length ratio of four tarsal segments is 1.3:1:1.1:3.4 (Fig. 8D). Hindlegs (5.58 mm), length ratio of femur and tibia = 1.6:1; length ratio of four tarsal segments is 1.4:1:1.2:3.4 (Fig. 8E). Mesonotum, brown with a notable median longitudinal suture (Fig. 8F). Mesosternum, pale red with a square basisternum, broad furcasternum (Fig. 8G). Forewing and hindwing are similar to imago but more opaque and having more visible crossveins (Fig. 8H, I). ***Abdomen*.** Genitalia similar to those of imago but forceps and penes shorter and broader; forceps with total length = 1.0 mm, first segment = 0.53 mm, second segment= 0.40 mm and third segment = 0.07 mm; penes length = 0.7 mm (Fig. 8J–L). Abdomen brown and pale red dorsally, segment IX with notable lateral projections (Fig. 8M–O).

**Female imago.** (in alcohol, Fig. 9). ***Head*.** Eyes rounded, dark brown without dorsal eyes (Fig. 9A, B). ***Thorax*.** Forelegs (7.2 mm), length ratio of femur and tibia = 1.4:1; length ratio of four tarsal segments is 1:1:1:3.7 (Fig. 9C). Midlegs (6.4 mm), length ratio of femur and tibia = 1.2:1; length ratio of four tarsal segments is 1:1:1.2:2.8 (Fig. 9D). Hindlegs (7.2 mm), length ratio of femur and tibia = 1:1, length ratio of four tarsal segments is 1:1.2:1.1:2.6 (Fig. 9E). Mesonotum brown with a notable median longitudinal suture (Fig. 9F). Mesoternum pale red with rectangle basisternum, broad furcasternum (Fig. 9G). Forewing, 14 crossveins in stigmatic area; MA forked middle of wing; MP forked basally, 3 intercalaries between MP1 and MP2; CuA and CuP adjacent at base (Fig. 9H). Hindwing rounded, leading margin slightly concave, with clear crossveins, 7 crossveins between MA and MP (Fig. 9I). ***Abdomen*.** Tergites brown, sternites pale red, sternites VIII-IX brown (Fig. 9K–N). Subanal plate brown with shallow median cleft (Fig. 9J). Subgenital plate weakly developed, pale, with shallow median emargination (Fig. 9N)

***Eggs.*** (dissected from female imago). Length 175 µm, width 125 µm; oval shape, chorionic surface with small protuberances, half of egg covered with helmet-shaped polar cap (Fig. 11A); many KCT around egg body (Fig. 11B); 1 or 2 tagenoform-type of micropyles at centre (Fig. 11C).

###### Distribution.

Kanchanaburi and Prachuap Khiri Khan Provinces, Thailand.

###### Remarks.

The larvae of *Vietnamella thani* are widely distributed in Thailand. They inhabit fast-flowing streams (Fig. 10A) but have never been reported in southern Thailand. *Vietnamella thani* have notable outer pairs of projections on head without serrated spines that differ from *Vietnamella* sp. B. The larvae show colour variations and can be greenish, yellow or brown. The imaginal stages of *V. thani* have similar characters to those of *V. sinensis* but lack the longitudinal vein on the stigmatic area of the forewing and they have less crossveins between Sc and RA on the hindwings (Table 4). The egg structure is covered by a membrane, which leads to unclear sculpturing of the surface, especially in the posterior part of the egg. Thus, the egg from this study showed little difference from the egg structure of *V. sinensis*.

### Molecular analysis

**Figure 12.**
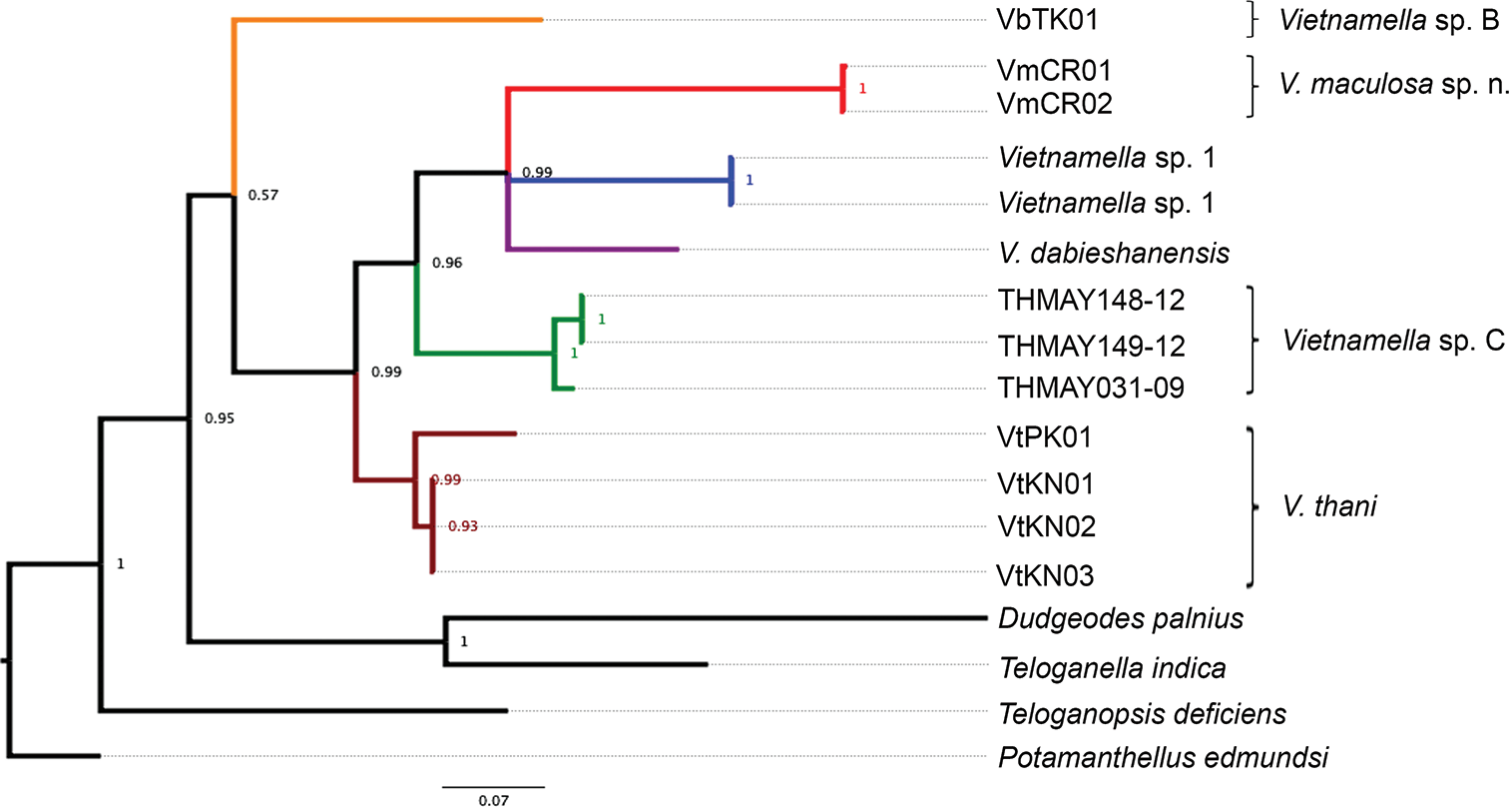
Bayesian interference of Ephemerelloidea. The COI phylogenetic construction of *Vietnamella* and sister groups with the percentages indicating branch probability support. *Potamanthellus edmundsi* was used as the outgroup.

The phylogenetic tree of vietnamellid mayflies and the other families of Ephemerelloidea was constructed from 658 bp of COI sequences by Bayesian interference. The results show that Vietnamellidae is clearly separated from the others. Six clades can be recognized within Vietnamellidae with high posterior probability value support for the morphospecies: *Vietnamella maculosa* sp. nov., *Vietnamella* sp. B, *Vietnamella* sp. C, *Vietnamella* sp. 1, *V. dabieshanensis* and *V. thani* (Fig. 12) Moreover, K2P genetic distance was analyzed to confirm species delimitation. The intraspecific genetic distances vary between 0–6.7 % whereas interspecific distances are very high, ranging from 16–31% (Table 2). The phylogenetic tree and K2P value result confirm that there are four different species of *Vietnamella* in Thailand.

## Discussion

Comparison of the larvae of *Vietnamella*, including *V. thani*, *V. sinensis*, *Vietnamella* sp. A, *Vietnamella* sp. B and *Vietnamella maculosa* sp. nov., is presented in Table 3. *Vietnamella ornata*, which was previously reported from Yunnan, China is not included because its larvae are still unknown (Tshernova 1972). The comparisons showed that a major character, the serration of the outer projection on the head, can distinguish *Vietnamella* into two groups (serration and non-serration groups).The non-serration group consists of *V. thani* and *Vietnamella maculosa* sp. nov., which is most similar to *V. sinensis*. They have a second segment of the maxillary palp that is longer than the other segments in *V. sinensis* but is of medium or nearly equal length in *V. thani* and *Vietnamella maculosa* sp. nov. The serration group includes *Vietnamella* sp. A (India) and *Vietnamella* sp. B (Thailand).

**Table 3.** Comparison of larval characteristics of known *Vietnamella* species.

Characters	*V. thani*	*V. sinensis*	*Vietnamella maculosa* sp. nov.	*Vietnamella* sp. A	*Vietnamella* sp. B
Maxillary palp segment ratio	1.3:1.3:1	1:1.6:1	1.3:1.2:1	1:0.9:0.7	1.3:1:1.1
Outer pair of projections on head	Without serration	Without serration	Without serration	With serration	With serration
Median ridge projection of abdominal terga	Pair: I-IX	Pair: I-X	Pair: I-X	Pair: II–IX	Pair: II–VI, VIII–X; single: VII
Posterolateral projection on tergite X	Less developed	Moderately developed ^a^	Well developed	Moderately developed ^b^	Moderately developed
Distribution	Vietnam, Thailand, China	China	Thailand	India	Thailand

^a^Hu et al. (2017). Definition based on fig. 1A, p. 383. ^b^Selvakumar et al. (2018). Definition based on fig. 1, p. 995.

**Table 4.** Comparison of adult characteristics of known *Vietnamella* species.

**Characters**	***V. thani* (imago)**	***V. sinensis* (imago)**
Stigmatic area of forewing	Not divided by longitudinal vein	Divided by longitudinal vein
Penes	Slender, shallow median cleft	Slender, shallow median cleft
Subgenital plate	Convex	Slightly convex
Hindwing	8 or 9 crossveins between Sc and RA	12 crossveins between Sc and RA

Egg structure of *Vietnamella* species has a similar pattern of a polar cap covering half of the egg chorion; however, we found little difference between the three species known at that stage: *V. maculosa* sp. nov., *V. thani* and *V. sinensis*. In addition, *V. maculosa* sp. nov. has a rod-shaped KCT that is different from that in *V. thani* and *V. sinensis* which have oval-shaped KCT. The chorionic surface of *V. maculosa* sp. nov. and *V. thani* have a protuberance which is smaller than in *V. sinensis* (Hu et al. 2017, fig. 6). From our results, it appears that the egg structure can be useful for species identification at least in the three *Vietnamella* species investigated. Considering other ootaxonomic investigations, egg structure of some mayflies can be used for identification at the species level (Sivaramakrishnan and Venkataraman 1987; Ubero-Pascal and Puig 2007).

**Figure 13.**
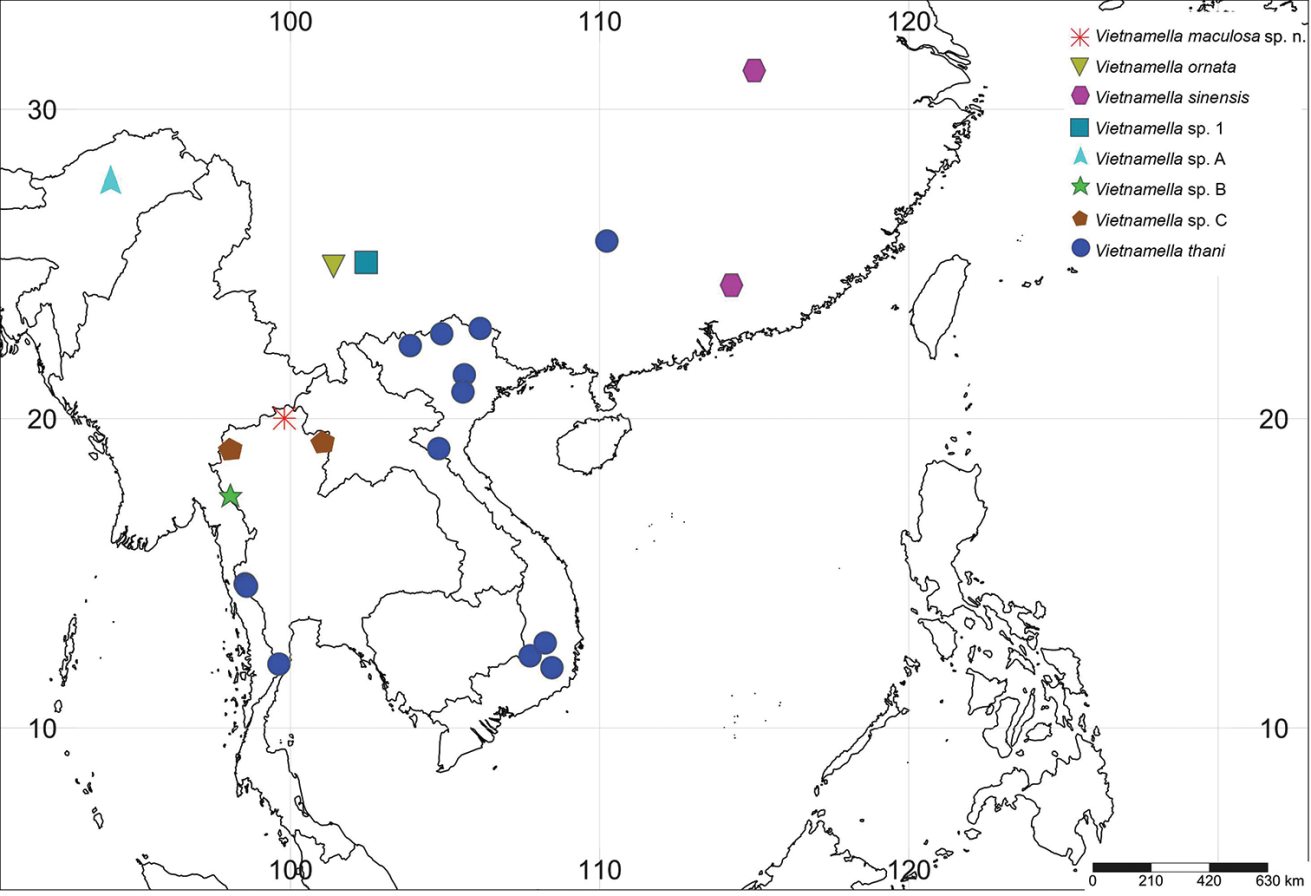
Distribution map of *Vietnamella* in the Oriental region. Each different shape represents a different species. Our specimens are only distributed in Thailand and including the data of *Vietnamella* sp. C from the BOLD system. Outside of Thailand distribution of *Vietnamella* is based on previous records including *V. ornata* (Tshernova, 1972), *V. sinensis* (Hu et al. 2017), *Vietnamella* sp. 1 (Tang et al. 2014), *Vietnamella* sp. A (Selvakumar et al. 2018) and *V. thani* (Hu et al. 2017 and unpublished data).

Our phylogeny indicated the existence of six different species of *Vietnamella* in the Oriental region, with four of them found in Thailand. Only one species, *V. thani*, can be found in western Thailand. Surprisingly, three species distributed in northern Thailand are revealed here by molecular analysis. Herein, only *V. maculosa* can be described as new species. However, we suppose that *Vietnamella* sp. B and *Vietnamella* sp. C are putative new species which could be formally described when more material becomes available. Although our molecular phylogenetic result clearly showed species delimitation in Thailand, there are still ambiguities in other areas where COI sequence or morphological data are incomplete. Thus, we infer that the sequences of *Vietnamella* sp. 1 (KM207084.1; KM244655.1) may belong to *V. ornata* because their specimens were collected near the type locality (Fig. 13) (Tang et al. 2014). *Vietnamella* sp. A was recently reported from India and the authors suspect it can represent the unknown larvae of *V. ornata* (Selvakumar et al. 2018). In addition, *V. dabieshanensis* (HM067837.1) is now considered as a junior synonym of *V. sinensis* (Hu et al. 2017) and this sequence likely refers to this species.

Although ambiguous classifications of Vietnamellidae still remain, our results allow us to conclude that at least four valid species (*V. thani*, *V. ornata*, *V. sinensis* and *V. maculosa* sp. nov.) exist, as supported by our morphological and molecular analyses. The findings of this study also extend the species diversity, imaginal description and phylogeny for future considerations of the Vietnamellidae.

## Supplementary Material

XML Treatment for Vietnamella maculosa

XML Treatment for Vietnamella

XML Treatment for Vietnamella thani
